# Treatment burden in multiple long-term conditions: a mixed-methods study protocol

**DOI:** 10.3399/BJGPO.2023.0097

**Published:** 2023-09-20

**Authors:** Rachel Johnson, Anastasiia G Kovalenko, Thomas Blakeman, Maria Panagioti, Michael Lawton, Shoba Dawson, Polly Duncan, Simon DS Fraser, Jose M Valderas, Simon Chilcott, Rebecca Goulding, Chris Salisbury

**Affiliations:** 1 Centre for Academic Primary Care, Bristol Medical School, University of Bristol, Bristol, UK; 2 Division of Population Health, Health Services Research and Primary Care, The University of Manchester, Manchester, UK; 3 School of Primary Care, Population Science and Medical Education, Faculty of Medicine, University of Southampton, Southampton, UK; 4 Centre for Research in Health Systems Performance (CRiHSP) and Division of Family Medicine, Department of Medicine, Yong Loo Lin School of Medicine, National University of Singapore, Queenstown, Singapore

**Keywords:** multimorbidity, primary health care, young adult, primary healthcare, general practice

## Abstract

**Background:**

Treatment burden represents the work patients undertake because of their health care, and the impact of that effort on the patient. Most research has focused on older adults (aged >65 years) with multiple long-term conditions (multimorbidity) (MLTC-M), but there are now more younger adults (aged 18–65 years) living with MLTC-M and they may experience treatment burden differently. Understanding experiences of treatment burden, and identifying those most at risk of high treatment burden, are important for designing primary care services to meet their needs.

**Aim:**

To understand the treatment burden associated with MLTC-M, for people aged 18–65 years, and how primary care services affect this burden.

**Design & setting:**

Mixed-methods study in up to 33 primary care practices in two UK regions.

**Method:**

The following two approaches will be used: (i) in-depth qualitative interviews with adults living with MLTC-M (approximately 40 participants) to understand their experiences of treatment burden and the impact of primary care, with a think-aloud aspect to explore face validity of a novel short treatment burden questionnaire (STBQ) for routine clinical use in the initial 15 interviews; (ii) cross-sectional patient survey (approximately 1000 participants), with linked routine medical record data to examine the factors associated with treatment burden for people living with MLTC-M, and to test the validity of STBQ.

**Conclusion:**

This study will generate in-depth understanding of the treatment burden experienced by people aged 18–65 years living with MLTC-M, and how primary care services affect this burden. This will inform further development and testing of interventions to reduce treatment burden, and potentially influence MLTC-M trajectories and improve health outcomes.

## Introduction

Multiple long-term conditions (multimorbidity) (MLTC-M) is defined as the existence of two or more long-term conditions. It affects approximately one in four of the UK population, is associated with reduced quality of life and increased hospital admissions,^1,2^ and accounts for more than half of the costs of primary and secondary care.^3^ MLTC-M is more prevalent, and occurs at a younger age, in more deprived areas, contributing to health inequalities.^3–5^ MLTC-M disproportionately affects those living in areas of socioeconomic deprivation and minority ethnic groups.^6^ Most research on MLTC-M has included patients aged >65 years, however, almost one-third of people with four or more conditions are under this age.^7^

Treatment burden represents the work that patients undertake because of their health care, and the impact of that effort on patients.^8,9^ Younger populations may experience different challenges that affect their treatment burden. Interventions focused on younger populations have the potential, through addressing treatment burden at an earlier stage, to influence trajectories of MLTC-M.^10^

UK National Institute for Health and Care Excellence (NICE) MLTC-M guidance^11^ recognises the need to reduce treatment burden.^12^ However, no existing interventions have shown convincing reduction of treatment burden for people living with MLTC-M.^13–15^

Few qualitative studies have investigated the experience of treatment burden for people with MLTC-M in primary care.^16,17^ A recent systematic review of the impact of interventions on patient-reported burden of treatment included 11 studies, only one of which focused on people living with MLTC-M.^14^ Available measures of treatment burden are too time-consuming to be used in routine clinical practice to identify patients at risk of being overburdened by the demands of their health care. The multimorbidity treatment burden questionnaire (MTBQ) is a validated measure developed to capture the effort required to manage MLTC-M.^7^ It has been used to evaluate treatment burden in two UK surveys, largely focused on people in older age groups, and more affluent and minimally diverse populations.^7,18^ Each of these studies has evaluated the performance of a different single-item measure alongside the MTBQ; these had limited sensitivity and positive predictive value.^18,19^ Practical ways of measuring treatment burden in routine primary care practice would be valuable, enabling identification of people who are more likely to be overburdened.

To the authors' knowledge, no studies have explored the impact the organisation of primary care services has on the treatment burden experienced by people with MLTC-M. This protocol describes a mixed-methods study. The overarching aim is to understand the treatment burden associated with MLTC-M for people aged 18–65 years, and how primary care services affect this burden, in order to inform service design. The study will:

1a) explore, in-depth, their experiences of treatment burden and its impact;

1b) explore the face validity of the STBQ;

2a) examine the factors associated with treatment burden for adults living with MLTC-M;

2b) test the validity of the STBQ for routine clinical use.

## Method

**Figure 1.**
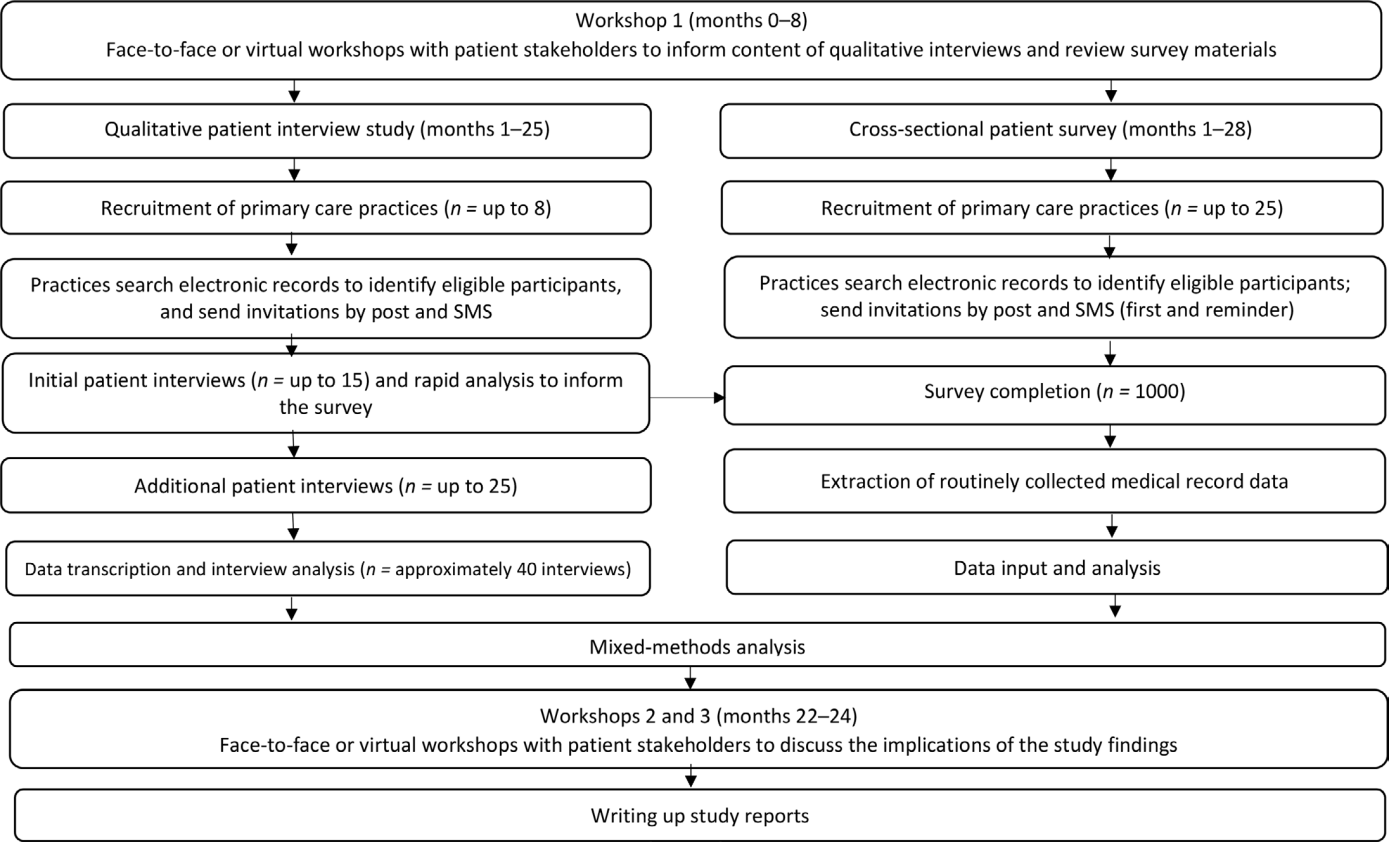
Study flowchart. SMS = short message service

It will be a concurrent mixed-methods study, including qualitative and quantitative components, and stakeholder engagement (Figure 1).

### Theoretical framework

The cumulative complexity model^20^ is used as a theoretical framework for the study. It describes the balance between the workload that an individual experiences because of their health care, and the capacity they have to manage that workload.

### Definition of multimorbidity

The 20-condition Cambridge Multimorbidity Score will be used to identify eligible participants.^4,21^ GP electronic record searches will be developed, based on the published code sets, to identify people with two or more of these 20 conditions.

Participants will be adults (aged 18–65 years) with two or more long-term conditions.

The following will be excluded: people with dementia; those lacking capacity to consent; people receiving palliative care; and nursing home or care home residents.

### Inclusivity

People from ethnic minority groups are likely to report poorer health outcomes and experiences of accessing health services than their White British counterparts.^6^ They are often under-represented in research, limiting the relevance and generalisability of results.

The study will seek to increase participation of people from ethnic minority groups and socioeconomically disadvantaged communities. All participant materials will be translated and back-translated into commonly spoken languages in the study areas. Interpreters will be available for interviews.

### Qualitative study

#### Design

Semi-structured qualitative interviews will be undertaken with adults living with MLTC-M exploring objectives 1 a,b.

#### Sampling

The qualitative study will recruit up to eight primary care practices across two geographical areas. Participants will be purposively sampled to achieve maximal variation in practice-level deprivation and rurality, patient age, sex, ethnic group, employment status, being a carer, and type of MLTC. Invitations will be sent to eligible patients identified by electronic record searches in participating practices. Interested people will contact the study team to arrange an interview in-person, by telephone, or video call. Fully informed consent will be taken at the time of the interview (written or audio-recorded).

#### Data collection

Topic guides have been developed and piloted with input from the patient and public involvement (PPI) group. In-depth interviews will focus on patients’ experiences of MLTC-M burden and their capacity to manage the workload. The following will be explored: how different health conditions interact; how the experience of burden changes with time and circumstances; how patients navigate primary care services; and the impact of health services on MLTC-M burden and capacity. Interviews will be audio-recorded, professionally transcribed, anonymised, and managed in NVivo (version 12).

Up to 15 initial interviews will explore participants’ thoughts about the STBQ. Participants will be asked to think aloud^22^ as they complete the measure, including commenting on the layout and wording, and discussing the reasoning behind their questionnaire responses. These interviews will be carried out in blocks of 3–5. At the end of each block, the data will be reviewed and the questionnaire modified.

Participating patients will be offered a £25 shopping voucher.

#### Analysis

The analysis will involve two stages.

*Objective 1 a,b*. Data analysis will be thematic,^23^ conducted by the interviewers, members of the research team, and up to two public contributors from the PPI group. Analysis will begin with line-by-line coding, followed by discussion to agree the coding frame. Transcripts will be coded by one researcher, and a randomly chosen sample will be reviewed independently by a second researcher. The researchers will initially identify themes, which will then be discussed with other members of the research team. Analysis will continue alongside data collection, allowing the topic guides to be modified to respond to findings. Up to two members of the PPI group will be invited to contribute to the analysis by (i) being involved in a facilitated discussion in which codes are developed and researcher interpretations of the data are checked; (ii) using selected extracts from transcripts to sense-check, refine, and expand themes.

*Objective 1b*. Framework analysis^24^ will be conducted to analyse the think-aloud interviews. The researchers will summarise the data within a framework matrix, based on the different aspects of the questionnaire. The final version of the STBQ will be used in the survey.

Sample size and participant recruitment will be determined based on the concept of information power.^25^ The analysis is informed by established theory, interviews will be focused on the research questions, and it is anticipated that participants will have rich experiences relevant to the research question. These factors will increase the information power of the sample. Sufficient information power will be achieved when the sample is deemed to have addressed the study’s research questions (the research team estimates 30–40 interviews).

### Quantitative study

#### Design

A cross-sectional patient survey and analysis of linked routinely collected GP record data will be undertaken, addressing objectives 2 a,b.

#### Sampling

The quantitative study will sample up to 25 primary care practices across two geographical areas in England, aiming to recruit 50% of practices from Index of Multiple Deprivation (IMD) deciles 1–5 (with one being most deprived and 10 least deprived) and at least six practices in deciles 1–3. Practices will run electronic searches to identify eligible patients, and will invite a random sample of up to 500. Sample size calculation is presented in Table 1.

**Table 1.** Power for total sample size of 1000, baseline risk of high burden 20% and risk of high burden 30% in group with characteristic of interest

	Prevalence of patient characteristic of interest^a^						
Power	0.2	0.25	0.3	0.35	0.4	0.45	0.5
	84.4%	89.2%	92.1%	93.8%	94.9%	95.4%	95.6%

^a^For example, patient living in a deprived area.

#### Data collection

##### i. Survey

The measures included in the survey are described in Table 2.

**Table 2.** Survey measures

Concept	Measure	Description
Sociodemographic data	Age, sex, ethnic group, and employment status	Participants are asked to describe: age sex ethnic group (selecting from the list provided here: https://www.ethnicity-facts-figures.service.gov.uk/style-guide/ethnic-groups) employment status
Health status	a. PROMIS-10^26,27^b. Self-reported long-term conditions	a. A validated 10-item person-centred measure of health and functioning for people with long-term conditions. The questions are a better fit for the study's purposes than those included in, for example, the SF-12.b. One question asks the participant to list the conditions they believe they have, which have lasted or will last longer than 6 months.
Treatment burden	a. The Multimorbidity Treatment Burden Questionnaire (MTBQ)^7^b. Novel short treatment burden questionnaire (STBQ)	a. The MTBQ is a concise, simply worded set of questions to measure treatment burden in people with MLTC-M. In this study, the 13-item questionnaire will be used.b. Building on previous work to develop a single-question screening measure for treatment burden,^18^ the STBQ has been developed with PPI input. It includes two questions: one to screen for high treatment burden, and one to understand what they find difficult from a range of options. The STBQ has been developed for use in clinical practice, rather than as a research tool. It may be revised in response to feedback from initial qualitative interviews.
Primary care experience	PCPCM^28^	The PCPCM focuses on the patient’s access to care, relationship with the doctor or practice, and ability to reach health outcome goals. It comprises 11 items that form an evaluation of access, continuity, comprehensiveness, coordination, advocacy, family and community context, and goal-oriented care.
Health literacy	SILS^29^	A validated single-item screening instrument, designed to identify patients with limited reading ability who need help reading health-related materials.
Healthcare use	Healthcare use^30^	Five questions will be included, adapted from Salisbury and colleagues,^30^ asking whether participants have recently stayed at an NHS hospital, visited A&E, and taken time off work to attend hospital and GP appointments.
Other		The survey may additionally include a small number of questions from other validated questionnaires if it is apparent from stakeholder work or initial qualitative work that any issues create treatment burden for patients which are not already included in the other data sources proposed.

A&E = accident and emergency department. MLTC-M= multiple long-term conditions (multimorbidity). MTBQ = Multimorbidity Treatment Burden Questionnaire. PCPCM = Person-Centered Primary Care Measure. PROMIS-10 = Patient-Reported Outcomes Measurement Information System. SF-12 = 12-Item Short Form Health Survey. SILS = Single Item Literacy Screener. STBQ = short treatment burden questionnaire.

##### ii. Medical records

Survey responders will be asked to consent to access to their medical records. For consenting participants, the following data will be collected and linked to the survey data for analysis: age, sex, individual-level deprivation, number and type of long-term health conditions, number of prescribed medications, and number and type of consultations in general practice. In addition, anonymised data will be collected on the age, sex, deprivation level, and number of long-term conditions of all patients invited to complete the survey, facilitating comparison with the responder sample.

### Data management and analysis

Data will be managed in a REDCap database and analysed using Stata (version 17). Unclear questionnaire data will be treated as missing. Descriptive analyses will report MTBQ, PROMIS-10 (Patient-Reported Outcomes Measurement Information System), and PCPCM (Person-Centered Primary Care Measure) by the other variables of interest.

#### Regression analyses

The association of MTBQ scores will be investigated with the variables of interest. The following three types of regression models will be explored: logistic regression where MTBQ scores are dichotomised into those with and without high burden; ordinal logistic regression where the MTBQ score is categorised into different levels of burden; and linear regression with the global MTBQ scores as a continuous measure. Initially, each of the variables of interest will be assessed in univariate models and then multivariable models will be built using stepwise methods. Multicollinearity will be assessed using variance inflation factors and non-linear associations will be considered for numeric variables. Depending on missing data, complete-case analyses and also imputed analyses will be carried out. The results from the linear regression will be presented as primary results with the rest as sensitivity analyses dependent on testing the assumptions in the regression models.

More details are available in Supplementary material S1.

#### STBQ validation

Different versions of the STBQ will be explored through inter-item correlations, internal consistency (Cronbach’s alpha), and comparison of the association between the STBQ with high treatment burden as measured by the MTBQ using the receiver operator characteristic curve and diagnostic parameters: sensitivity, specificity, and positive and negative predictive values. The aim is to achieve the shortest possible questionnaire that has a high sensitivity.

### Stakeholder engagement

Three workshops will be held in the two study sites. An early workshop will engage a diverse group of people living with MLTC-M in discussions about the content of the interviews and survey. In two late workshops the authors will engage with stakeholders representing patients, primary healthcare services, commissioners, and policymakers to identify the implications of the research findings.

### Patient and public involvement

The PPI group of eight members with lived experience of MLTC-M contributed to the development of the research questions, the study protocol, study documentation, and the design of the survey and qualitative topic guide. They will be involved in the analysis of the qualitative data, interpretation, and dissemination of the study findings.

## Discussion

This study will use qualitative interviews with patients, and a cross-sectional patient survey linked to routine data, to understand treatment burden experienced by people aged 18–65 years living with MLTC-M, and the ways in which the organisation of primary care services affects this burden. Through the qualitative research, the cumulative complexity model will be used to understand how and why treatment burden affects younger people, and how workload and capacity interact.^20^ Through the cross-sectional survey with linked routine data, this research will identify the factors associated with treatment burden, and associations between treatment burden and quality of life. Finally, the study will seek to validate a short treatment burden measure for use in routine clinical care. Practical recommendations will be produced for how primary care services can reduce treatment burden experienced by people aged 18–65 years living with MLTC-M. This will lead to further development and testing of interventions to reduce MLTC-M burden, with the potential to influence MLTC-M trajectories and improve health outcomes.

## Data

The data from this study will be owned by the University of Bristol. On completion of the study, the data will be analysed and tabulated and a Final Study Report prepared. The Final Study Report (and/or research papers) will be made openly and freely available via the University of Bristol Research Portal, and also on the project website.
